# Bioprospecting Desert Plants for Endophytic and Biostimulant Microbes: A Strategy for Enhancing Agricultural Production in a Hotter, Drier Future

**DOI:** 10.3390/biology10100961

**Published:** 2021-09-26

**Authors:** Qiuwei Zhang, James F. White

**Affiliations:** Department of Plant Biology, School of Environmental and Biological Sciences, Rutgers University, New Brunswick, NJ 08901, USA

**Keywords:** endophytes, biostimulant microbes, plant–microbe interactions, climate change, desert plants

## Abstract

**Simple Summary:**

Endophytes are microbes that live inside plants without causing negative effects in their hosts. All land plants are known to have endophytes, and these endophytes have the capacity to be transferred between plants. Taking endophytes from desert plants, which grow in low-nutrient, high-stress environments, and transferring them to crop plants may alleviate some of the challenges being faced by the agricultural industry, such as increasing drought frequency and rising opposition to chemical use in agriculture. Studies have shown that desert endophytes have the capacity to increase nutrient uptake and increase plant resistance to drought and heat stress, salt stress, and pathogen attack. Currently, the agricultural industry focuses on using irrigation, chemical fertilizers, and chemical pesticides to solve such issues, which can be extremely damaging to the environment. While there is still a lot that is unknown about endophytes, particularly desert plant endophytes, current research provides evidence that desert plant endophytes could be an environmentally friendly alternative to the conventional solutions being applied today.

**Abstract:**

Deserts are challenging places for plants to survive in due to low nutrient availability, drought and heat stress, water stress, and herbivory. Endophytes—microbes that colonize and infect plant tissues without causing apparent disease—may contribute to plant success in such harsh environments. Current knowledge of desert plant endophytes is limited, but studies performed so far reveal that they can improve host nutrient acquisition, increase host tolerance to abiotic stresses, and increase host resistance to biotic stresses. When considered in combination with their broad host range and high colonization rate, there is great potential for desert endophytes to be used in a commercial agricultural setting, especially as croplands face more frequent and severe droughts due to climate change and as the agricultural industry faces mounting pressure to break away from agrochemicals towards more environmentally friendly alternatives. Much is still unknown about desert endophytes, but future studies may prove fruitful for the discovery of new endophyte-based biofertilizers, biocontrol agents, and abiotic stress relievers of crops.

## 1. Introduction

Deserts present unique challenges for plant growth and survival. Infrequent and unpredictable precipitation, combined with high rates of evapotranspiration, results in dry surface soils with high salt concentrations [1]. In addition to being salty, desert soils are often nutrient poor, lacking in biologically accessible nitrogen and phosphorus [2,3]. The formation of “desert pavements” on top of desert soils reduces water penetration [4] and deters plant growth [5]. Air temperatures in deserts can fluctuate dramatically, sometimes by as much as 38 °C in the span of a day [6]. Overall, deserts are hostile environments for most plants, yet certain plant families have evolved to survive in deserts.

In addition to physical adaptations (such as CAM photosynthesis and modified leaf structures) that allow them to thrive in dry, nutrient-poor soils, desert plants also take advantage of microbial endophytes. Endophytes are defined as microbes that colonize plant tissues without causing apparent harm to their hosts [7]. They can be found in all land plants and are often required to maintain the health of their plant hosts [8]. Some endophytes are culturable in vitro, but many cannot be cultured outside of their specific host tissues [9,10]. Endophytes that can be cultured in vitro can be transferred from their source host into a compatible secondary host to provide similar benefits [11,12]. The agriculture industry in particular uses endophyte inoculants for commercial purposes as biostimulants and biocontrol agents [13,14,15,16]. However, despite the commercial and scientific interest in endophytes, not much research has been performed on the endophytes of wild plants, and even less research has been performed on the endophytes of wild desert plants.

Desert plants may serve as an untapped source of novel endophytes for use in agriculture, especially in arid farming areas where water and soil nutrients are at a premium. Desert endophytes may also have applications worldwide, as global climate changes increasingly subject croplands to abiotic stressors common in deserts. Rising CO_2_ levels are expected to result in longer and more severe instances of drought, including instances of agricultural drought, which is characterized by decreases in soil moisture that negatively affect crop growth [17,18,19]. Likewise, instances of abnormally heavy rains and flooding are also expected to increase [20,21]. In addition, calls for reduced applications of chemical fertilizers, which are known to contaminate water sources by means of leaching and runoff [22,23,24], will lead to reduced soil nutrient levels that will require more efficient uptake by crops. The application of desert plant endophytes to crops may serve to alleviate some of the problems that the agricultural industry must contend with, both currently and in the future, though special care should be given to ensure that these endophytes will synergize with the new hosts’ native microbiomes. A switch from agrochemicals to microbe-based alternatives would also have the added benefit of public support, as evidenced by the increasing consumer demand for organic-certified foods [25,26,27] and the increasingly negative perceptions of chemical pesticides [28,29,30].

In this review, we detail what is currently known about desert plant endophytes and explore their possible commercial and environmental benefits.

## 2. Microbial Endophytes Present in Desert Plants

Desert soils are unique due to their sandiness, high salt concentration, low nutrient content, and low surface moisture. Lundberg et al. [31] demonstrated that plant root microbiomes are influenced by the soils in which they are grown, so the uniqueness of desert soils may result in the natural accumulation of endophytes that are able to help desert plants survive the unique challenges of desert living. Indeed, work by Colemann-Derr et al. [32] and Desgarennes et al. [33] on the microbiomes of agaves demonstrates changes to the core agave microbiome during dry seasons, lending credence to the idea that desert plants may be able to obtain useful endophytes under stressful conditions. These endophytes may then be cultured in vitro and inoculated onto new hosts.

In the first part of this review, we seek to analyze common genera of endophytes that are present in many different desert species to gain a better understanding of what types of microbes are common in desert plants. Identification of the most common genera may help guide researchers who are interested in bioprospecting desert plants for beneficial endophytes. However, it is important to note that the genera mentioned below are composed only of culturable endophytes. There may be many more genera that are common to desert plant microbiomes but are unculturable outside their native host tissue; therefore, they are omitted, as unculturable microbes are of little commercial use as biostimulants and biocontrol agents despite their potential benefits. In addition, it must be mentioned that the diversity of culturable endophytes in this meta-analysis may not reflect the true diversity of culturable endophytes due to the lack of research on the topic.

It is also possible that the ability of desert endophytes to confer benefits to their hosts is not determined by their taxonomy, but rather by the expression of certain genes related to biotic and abiotic stress resistance. A meta-analysis on transcriptomic and metabolomic studies of desert endophytes would help tease out important genes that are common between beneficial desert endophytes; however, most published research concerning desert endophytes focuses on categorizing the beneficial effects of the endophytes themselves without delving further into the transcriptome or metabolome of the endophyte, making it hard to ascertain if such groupings exist. More studies should be performed on this topic and further meta-analyses could and should be performed once more information is available.

### 2.1. Bacterial Endophytes

**Figure 1:**
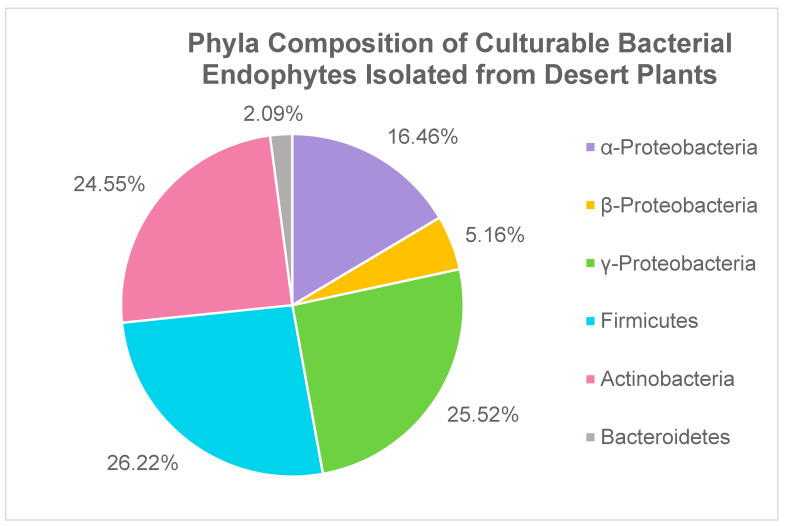
Culturable bacterial isolates from the meta-analysis categorized by phyla. Individual isolates from 12 studies were pooled together and categorized based on which phylum they belonged to. References: [36,37,38,39,40,41,42,43,44,45,46,47].

A meta-analysis of 11 studies aimed at identifying culturable bacterial endophytes of various desert plants revealed that all isolates belonged to one of four major phyla: Proteobacteria, Firmicutes, Actinobacteria, and Bacteroidetes. Out of a total of 717 bacterial isolates identified, 47.14% belonged to the phylum Proteobacteria, 26.22% belonged to the phylum Firmicutes, 22.55% belonged to the phylum Actinobacteria and 2.09% belonged to the phylum Bacteroides (Figure 1). This phylum distribution of desert bacterial endophytes appears to be consistent with previous reports on endophyte diversity in non-desert plants [34,35].

Within each phylum, certain genera appear multiple times (Table 1). For phylum Proteobacteria, Pseudomonas is the most common genus, comprising 15.68% of all Proteobacteria isolates, but other genera, such as *Acinetobacter* and *Gluconobacter*, also appear at similar frequencies. For phylum Firmicutes, *Bacillus* is the most common and most dominant genus, comprising 78.72% of all Firmicutes isolates. For phylum Actinobacteria, *Microbacterium* is the most common genus, comprising 51.70% of all Actinobacteria isolates. Though *Sphingobacterium* appears to be the most common genus for phylum Bacteroides, it is uncertain whether it is a common desert endophyte due to the very limited number of culturable isolates belonging to phylum Bacteroides.

### 2.2. Fungal Endophytes

**Figure 2:**
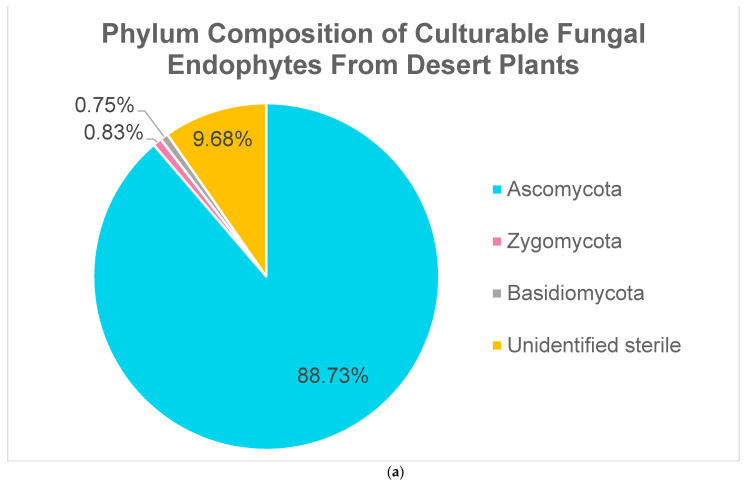

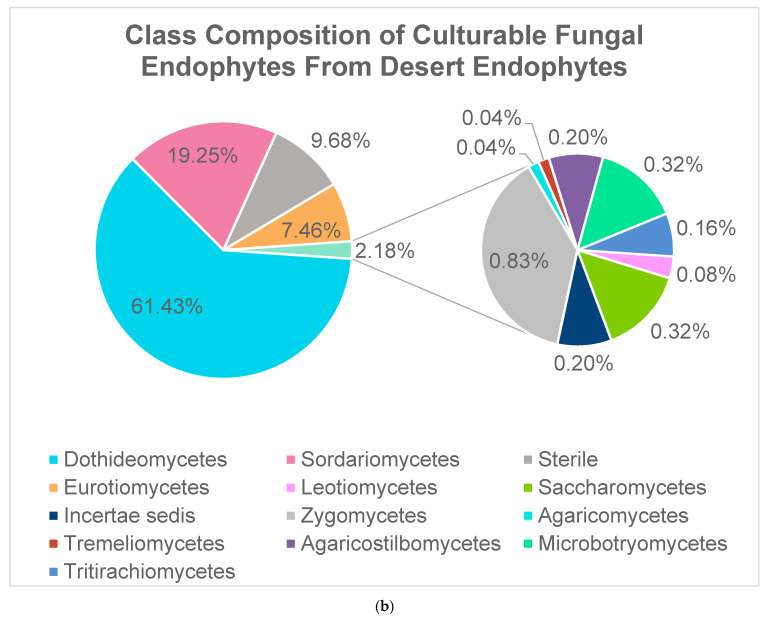
Culturable fungal endophytes from the meta-analysis categorized by (**a**) phylum and (**b**) class. Individual isolates from seven studies were pooled together and categorized based on which phylum or class they belonged to. References: [51,52,53,54,55,56,57].

A meta-analysis of seven studies aimed at identifying culturable fungal endophytes of various desert plants revealed that 88.73% of the isolates belonged to the phylum Ascomycetes, 9.68% were sterile forms, 0.83% belonged to the phylum Zygomycota, and 0.75% belonged to the phylum Basidiomycota (Figure 2a). A majority of ascomycete endophytes are members of the Pezizomycotina, with a few belonging to the Saccharomycotina and five with uncertain taxonomy. All of the Basidiomycete endophytes are members of the Agaricomycotina and Pucciniomycotina. All of the Zygomycete endophytes are members of the Mucoromycotina. These results are consistent with what was previously known about fungal endophyte diversity in plants [48]. The prevalence of Dothiomycetes, Sordariomycetes, and Eurotiomycetes is similar to previous reports on desert endophyte diversity [49,50].

Certain genera appear to dominate each class of fungi (Table 2). For class Dothideomycetes, *Alternaria* and *Phoma* are the most common genera, comprising of 30.75% and 28.68% of all Dothideomycetes isolates, respectively. For class Sordariomycetes, *Fusarium* is the most common and most dominant genus, comprising 27.01% of all Sordariomycetes isolates. For class Eurotiomycetes, *Penicillium* is the most common genus, comprising 71.28% of all Eurotiomycetes isolates. Due to the small number of isolates obtained for the other classes of fungi, it is unknown if any dominant genera exist.

Fungal endophyte diversity in desert plants appears to be lower than that of bacterial endophytes, as most isolates are members of the Ascomycota. That is to be expected, as Ascomycota is the largest phylum of kingdom Fungi. However, when compared to fungal endophyte profiles in the tropics, fungal endophyte profiles in deserts have lower diversity with higher overall tissue colonization rates [57,58]. Selection pressure for endophyte colonization may stem from a need for nutrients, which are not readily available in desert soils. Host-endophyte symbioses would be beneficial for both parties, as host plants would gain access to usable nitrogen and phosphorus, while the endophytes would gain access to sugars and protected habitats. Low endophyte diversity may be attributed to high UV radiation and low water availability in deserts [49].

While not reflected in the meta-analysis, one interesting pattern to note is the prevalence of dark septate endophytes (DSE) as fungal endophytes in arid regions. DSE are sterile root endophytes with melanized septate hypha; they do not belong to a specific class or genus but are categorized based on their physical characteristics. While DSE can be found across many ecosystems, they are most prevalent in high-stress environments such as deserts and alpine regions [59]. DSE may be particularly beneficial for plants living in arid conditions, as they are more common than true mycorrhizal fungi in high latitude polar regions [60] and desert grasslands [50,61]. While it is unknown exactly how much of the desert fungal endophyte population is made up of DSE, the meta-analysis of desert endophytic fungi revealed 244 sterile forms. If all 244 isolates happened to be DSE, they would make up 9.68% of all fungal endophytes in desert plants, making them the third most common group of endophytes after *Alternaria* and *Phoma* and equally as common as *Aureobasidium*.

### 2.3. Transfer of Endophytes between Desert Plants

It is important to note that endophytes are not confined to one host-they can be moved from one host to another. There are various ways of transferring endophytes between plants, separated into two categories: vertical transmission and horizontal transmission. Vertical transmission involves the passing of endophytes from mother to daughter plant via the inclusion of endophytes within and around the seed, while horizontal transmission involves the passing of endophytes between two separate individuals that may or may not be related and may or may not be of the same species.

Since endophytes are known to aid in host survival, endophyte transfer in deserts may be especially critical to plant survival due to the variety of abiotic stressors present in deserts that likely necessitate the presence of endophytes early in the plant life cycle. Vertical transmission from mother to daughter plant allows endophytes to be present before a seed has even germinated, which is important as some endophytes are known to increase the germination rate and germination speed of seeds [62,63]. Early colonization of plants by compatible endophytes likely increases the chance of seedling survival compared to non-colonized seedlings or seedlings colonized by non-compatible endophytes. However, this method of endophyte transfer may not be enough to ensure the efficient reproduction and spread of the endophyte itself, depending on the germination rate of seeds. For instance, desert agave seeds have an astronomically low rate of establishment—approximately 1 in 1,200,000 seeds end up becoming established plants [64]. Endophytes exclusive to desert agave and other low-seedling-establishment plants may be pressured to evolve into generalists so they can colonize other hosts in case their current host does not survive.

As desert plants grow, their microbiomes may shift from mostly vertically transmitted endophytes to include more and more horizontally transferred endophytes. These horizontally transferred endophytes may come from the roots of nearby plants or from more distant plants via herbivores. When feeding on desert plants, herbivores will end up ingesting endophytes along with vegetative tissue. Those endophytes may remain in the mouth and be passed to new hosts via mechanical damage [65], or may travel through the gut and be passed along via droppings [66,67]. While it is known that microbiome composition of plants changes with age [31,68], it is unknown how the microbiome composition of desert plants changes with time and how much of a change there is. Hoffman and Arnold [69] showed high host generalism amongst desert endophytes, which they hypothesized is due to the cost–benefit advantage of being able to escape the harsh desert environment by colonizing and hiding inside of a variety of hosts. This may point to a high degree of microbiome composition change over time, but without more research, it is impossible to tell for sure.

This host generalism of desert endophytes makes them a prime candidate for use in crops, as they are not restricted to a specific family or genus of host, but there may be downsides to this trait, as discussed later in this paper. Additionally, there are still gaps in our understanding of plant–microbe interactions, particularly with how the native host microbiomes of crop plants respond to environmental changes such as drought and how they respond to the introduction of non-native microorganisms under stressed and non-stressed conditions. Such factors should be considered and tested during the evaluation of an endophyte’s effect on a novel host, as slight changes in the environmental conditions may render a potentially beneficial endophyte useless—or worse, detrimental—to the host.

## 3. Nutrient Acquisition

Plants require a variety of nutrients to support their growth and development, the most important of which are nitrogen and phosphorus. Nitrogen and phosphorus are considered to be limiting factors for crop growth, hence why nitrogen and phosphorus fertilizers are commonly used in agriculture. However, heavy usage and reliance on these fertilizers has resulted in nonpoint pollution of surface waters via leaching and runoff [70,71]. Nitrogen and phosphorus pollution not only damages aquatic ecosystems [71,72,73], but also presents dangers to humans who rely on or come into contact with contaminated waters [71,74,75]. In addition, there are concerns regarding the production of chemical fertilizers and its impacts on the environment. For instance, chemical phosphorus fertilizers are produced from phosphate rock, but there are many questions pertaining to the sustainability of phosphate rock mining and the environmental impacts of the phosphate rock industry [76,77].

In order to reduce the impact of agriculture on the surrounding ecosystems, researchers have been trying to find environmentally friendly alternatives for supplying nitrogen and phosphorus to crops. One area of focus has been on endophytic microbes, which may be able to reduce a crop’s external nitrogen and phosphorus needs. Desert soils are naturally deficient in nitrogen and phosphorus, which may select for endophytes that allow their hosts to use available nutrients more efficiently or acquire them from novel sources.

### 3.1. Nitrogen

Even though nitrogen is abundant in the air as N_2_, plants cannot take up the atmospheric form of nitrogen and must obtain nitrogen from the soil in the form of nitrates. To bypass this reliance on soil nitrates, certain plants have evolved symbiotic relationships with nitrogen-fixing bacteria. The most well known of these plant–microbe associations occurs in legumes, where nitrogen-fixing rhizobia thrive in root nodules and provide their hosts with fixed nitrogen. In non-leguminous plants, where such intimate symbioses cannot be induced effectively or even at all, diazotrophic endophytes may serve as a suitable substitute for rhizobia. Diazotrophic endophytes have already been discovered in a wide variety of plants, including cottonwood and willow [78], sweet potato [79], and rice [80], but novel strains of diazotrophic endophytes may be found more commonly or in greater numbers in desert plants due to limited amounts of fixed nitrogen present in desert soils.

Many species and strains of the most commonly found desert plant bacterial endophytes (Proteobacteria, Actinobacteria and Firmicutes) have the capability to be nitrogen fixers [81]. The nitrogen-fixing ability of the most common genus of bacterial endophytes, *Bacillus*, has been well documented amongst certain species, namely *B. polymyxa, B. macerans,* and *B. azotofixans* [82,83,84]. The second most common genus of bacterial endophytes, *Pseudomonas*, has also been shown to have nitrogen-fixing members, namely *P. stutzeri* [85,86,87]. Less common endophytes, such as *Klebsiella* [88] and *Pantoea* [79] may possess nitrogen-fixing capabilities as well. Indeed, diazotrophic *Bacillus, Pseudomonas*, and *Klebsiella*, as well as *Acinetobacter, Cronobacter, Enterobacter, Enterococcus* and *Leuconostoc,* have been found in *Agave tequiliana* [42]. However, the most interesting and potentially beneficial diazotrophic endophytes are likely to be found in pioneer plants that colonize disturbed areas, particular areas with low amounts of soil.

Research performed on the roots of the cardon cactus *Pachycereus pringlei* revealed strains of *Bacillus* and *Klebsiella* that were able to fix nitrogen, even though the roots themselves contained no nodules [45,89]. The cardon cacti used in this study grow in volcanic areas where very little, if any, soil is present, so it is unlikely that they are relying on soil nitrates to fulfill their nitrogen needs. Another rock-colonizing cactus, *Mammillaria fraileana,* has also been shown to have nitrogen-fixing endophytes in the form of *Azobacter vinelandii* [41,90]. Like *P. pringlei*, *M. fraileana* grows in terrain where little to no soil is available, which limits the growth of other plants. This suggests that the two cacti obtain their nitrogen from other sources, likely their diazotrophic endophytes.

This begs the question—can biological nitrogen fixation from diazotrophic endophytes produce enough nitrogen to cover a plant’s nitrogen needs? There is some evidence suggesting that the answer is ‘yes’; biological nitrogen fixation with endophytes can make up for a certain percentage of soil nitrogen deficiencies. For instance, Puri et al. [91] inoculated spruce saplings with diazotrophic endophytes isolated from spruce trees growing in nitrogen-poor sub-boreal forests and grew them in nitrogen-poor soils; after a year of growth, it was revealed that that 17–56% of the total plant nitrogen of the saplings came from the atmosphere via biological nitrogen fixation. This effect also extended to a separate, non-spruce host. Likewise, inoculation with a diazotrophic *Klebsiella pneumoniae* from maize relieved nitrogen deficiency symptoms in wheat grown in nitrogen-deficient soils [88]. If highly efficient diazotrophic endophytes could be isolated from desert plants, it is likely that they can be transferred to crop plants to reduce their nitrogen requirements.

However, there is still some debate over whether or not increases in plant growth and development post-inoculation are strictly due to in planta nitrogen fixation by endophytes. Some argue that during photosynthesis, nitrogenases are inhibited by reactive oxygen species present in most plant tissues [92,93], but there are ways around this restriction on nitrogenase activity, via processes such as the upregulation of antioxidant production [94] or the upregulation of nitrogenase production genes under specific conditions [95,96,97]. Though a ^15^N_2_ incorporation experiment by Sevilla et al. [98] showed that endophytic *Acetobacter diazotrophicus* was able to fix nitrogen in planta, this may not be true of all endophytes. We would like to propose another explanation for how endophytes are able to transfer nitrogen to their hosts: a process termed the “rhizophagy cycle,” first proposed by Paungfoo-Lonhienne et al. [99]. We hypothesize that much of the nitrogen acquired by plants may be acquired in the roots via a process by which bacteria cycle between the soil and roots, bringing in nutrients from the soil which are then extracted from the microbes via reactive oxygen-mediated degradation within root cells [99,100]. We suspect that most of the biological nitrogen fixation in endophytes likely occurs during the free-living soil phase, when bacteria are not exposed to the reactive oxygen inside plant tissues; once they re-enter the host roots, they are subjected to host-produced superoxide and whatever fixed nitrogen they have acquired is absorbed by the host. We also propose another mechanism for intracellular nitrogen fixation for bacteria, where nitrogen fixation may occur within growing root hairs or other plant cells that are not active in photosynthesis, as the bacteria are protected from reactive oxygen [16,101]. Although much remains to be learned, diazotrophic endophytes of desert plants may participate in the rhizophagy cycle, as evidenced by a study on *Agave tequilana* [102]. Additional studies will need to be performed to determine the importance of the rhizophagy cycle as part of bacteria–plant interactions.

It is possible that endophytic fungi also participate in the rhizophagy cycle, as they can also form wall-less mycosomes inside plant tissues [100]. Martínez-Rodríguez et al. [42] has shown that endophytic *Diaporthe* sp. transfers organic nitrogen to its *Agave* host, though they note *Diaporthe* cells could not be recovered from *Agave* roots, suggesting that the fungus was degraded for its nitrogen. Soil yeasts have also been shown to participate in the rhizophagy cycle [103]. Research on DSE reveals that DSE have the capability to increase nitrogen and phosphorus content in various plants [104], however, they appear to be better at acquiring nitrogen from organic sources compared to inorganic sources [105,106,107]. It is unknown if desert DSE are participating in the rhizophagy cycle or transferring nitrogen to their hosts through other means. While DSE can break down organic nitrogen sources to provide their hosts with nitrogen [105,106], not much organic matter is available in deserts. More studies should be performed on the mechanisms by which endophytic fungi in desert plants can alter plant nitrogen acquisition.

### 3.2. Phosphorus

Just as with nitrogen, phosphorus is abundant, yet generally unavailable to plants. Large quantities of organic and inorganic phosphorus exist within agricultural soils, but much of it is immobilized in insoluble forms that are inaccessible by plants [108,109]. Insoluble phosphorus must first be transformed into a soluble form by soil microbes before they become available for plant usage. Many of these microbes can be found in the soil or in plant rhizospheres, though they are more common in rhizosphere soil than non-rhizosphere soil [110,111,112]. The proportion of phosphate-mobilizing microbes in the rhizosphere also changes depending on the type of plant, for instance, Katznelson et al. [112] found that cereals had lower numbers of phosphate-solubilizing microbes compared to other grasses and clover.

Phosphate-mobilizing microbes have also been found within plants as endophytes. Generally, phosphate-solubilizing bacterial endophytes belong to the Firmicutes or Proteobacteria phyla; examples include *Pseudomonas* [113], *Burkholderia* and *Rahnella* [114], *Bacillus* [115,116], and *Enterobacter* and *Pantoea* [116]. Most phosphate-solubilizing fungal endophytes are ascomycetes, though some may be basidiomycetes. Examples of ascomycete phosphate solubilizers include *Penicillium* [117,118], *Trichoderma* [119], *Aspergillus* [117], and *Fusarium* and *Humicola* [120]. An example of a basidiomycete phosphate solubilizer is *Piriformospora* [121].

All of the above genera, with the exception of *Humicola*, have been found as endophytic microbes in desert plants (Table 2).

Desert soils are generally rocky and poor in organic matter, so it is likely that desert plants rely on rock-bound phosphate for their phosphorus requirements. This is especially true for rock-colonizing plants which have no access to soils and must obtain their phosphorus from the rocks they grow on. Puente et al. [45,122] showed that several bacterial endophytes of *P. pringlei* were able to produce organic acids that weathered rocky substrates and extracted phosphates and other valuable minerals. The isolates were also able to grow on insoluble phosphate powder and solubilize it into orthophosphates that could be taken up by plants. Similarly, bacterial endophytes isolated from *M. fraileana* have also been shown to weather rocks and solubilize inorganic phosphate [41,90]. *P. pringlei* and *M. fraileana* are important pioneer plants that create soil from rocky substrates, so their endophytes may be more efficient at producing organic acids or produce an abundance of certain organic acids suited for rock substrates. Unfortunately, little information is available on endophytic fungi of desert pioneer plants. Since fungi are better at solubilizing phosphate than bacteria [123], fungal endophytes may contribute more to phosphorus acquisition and phosphate breakdown than bacterial endophytes. Without more research into the fungal endophyte composition of pioneer plants, it is impossible to say for sure.

Looking at non-pioneer desert plants, it seems that their DSE play a role in phosphorus acquisition. For instance, DSE from the genus *Aspergillus* isolated from the fourwing saltbush (*Atriplex canescens)* was found to solubilize inorganic phosphate [124]. Other DSE have been shown to enhance phosphorus acquisition by solubilizing various types of phosphate, including hardly solubilizable phosphates such as aluminum phosphate [117,125,126].

The solubilization of inorganic phosphates is important in terms of crop growth. Due to the usage of inorganic phosphate fertilizers, agricultural soils have accumulated a large amount of immobilized inorganic phosphorus that must be broken down prior to plant up-take [109]. In addition, there is evidence to suggest that soluble phosphates taken up by plants can become insoluble again once inside the plant, and that phosphate solubilizing bacteria are able to re-solubilize the phosphate in planta [114]. If such a process occurs in all plants, it would mean that endophytic phosphate-solubilizing bacteria are more important to plant phosphorus acquisition than rhizospheric phosphate-solubilizing bacteria. Desert plants, which have even less access to phosphorus than plants in other biomes, may harbor phosphorus-solubilizing endophytes that are more efficient at transforming inorganic phosphate to plant-available phosphorus than endophytes found in more nutrient-rich regions. This higher efficiency may apply to phosphate solubilization both inside and outside of plant tissues, which would reduce the amount of inorganic phosphate fertilizers needed for agricultural production and reduce inorganic phosphates leaching from soils.

It is important to note that in temperate areas, soil microbes quickly turn inorganic phosphate from fertilizers into organic phosphates [108], which are equally unavailable to plants. However, both bacteria and fungi are capable of breaking down organic phosphates. Matos et al. [115] showed that bacterial endophytes were capable of breaking down soy lecithin, an organic form of phosphate. DSE are able to readily break down organic phosphates [127,128], especially in the presence of organic nitrogen [106,129] so desert DSE may be able to function equally well in temperate agricultural regions. Not much is known about the ability of non-DSE fungal endophytes to break down organic phosphates, but they appear to have the capacity to produce phosphatases [117,126].

## 4. Effects on Abiotic Stress Resistance

As global temperatures and atmospheric CO_2_ levels continue to rise, agricultural regions are experiencing greater and greater frequencies of drought [18]. Plants growing under drought conditions must contend with a combination of water stress, heat stress, and salt stress, all of which negatively impact crop productivity and yields. The effects of drought on agriculture are compounded by water scarcity, especially in areas where water usage is mismanaged or where water sources are overexploited [130,131]. Access to freshwater for both agricultural and non-agricultural use has been dwindling in past decades due to increasing demand and decreasing supply, a problem which will only worsen as the global population continues to grow [132]. As the dominant consumer of freshwater throughout the globe, the agricultural industry must find sustainable ways to reduce its impact on water supplies. In addition, as the global population continues to grow, crops in developing nations may have to be planted and grown suboptimal conditions to meet increased food demands [133,134,135]. Finding ways to boost productivity under water-stressed and salt-stressed conditions would be a great boon for nations that are struggling to produce enough food to match demand.

Investment into research on desert plants and their microbiomes may provide solutions for both problems. Desert air temperatures can easily exceed 40 °C during the day and desert rainfall is notoriously low, forcing plants to constantly confront drought and heat stress. In addition, desert soils are high in salts, which can be just as deadly to plants as low water and high temperatures. Plants living in deserts are likely to naturally accumulate microbiomes that help them survive in hot, dry, salty environments, so transporting these desert endophytes into compatible crop hosts may increase the crops’ resistance to heat, drought, and high salinity. Adapting crops to harsher conditions would have the potential to both decrease the amount of water required for agriculture and boost crop yields in water-scarce regions [59,136,137].

### 4.1. Drought and Heat Tolerance

Though drought is defined as a deficiency in precipitation, drought and heat waves often occur together [138]. Water stress results in a variety of biochemical and physiological disturbances, including stomatal closure, altered enzyme activity, and membrane damage, all resulting in decreased CO_2_ assimilation and ATP synthesis [139]. Heat stress causes similar disturbances in CO_2_ assimilation and photosynthesis, but also includes other responses, such as increased number of reactive oxygen species and altered chloroplast metabolism [140,141]. Since photosynthesis becomes limited under water and heat stress, it is unsurprising that droughts and heat waves are known to decrease crop yields [17,18,19].

As droughts become more frequent and water scarcity becomes more common, the development of crop adaption mechanisms against drought stress and heat stress becomes ever more important. Currently, the agricultural industry utilizes a variety of practices to maintain crop productivity in the face of drought conditions, including the development of drought-resistant cultivars and the implementation of efficient irrigation systems. However, the introduction of drought- and heat-resistant microbiomes into crops has not really been considered, likely due to a lack of research on the topic.

Most of the available research on endophyte-based alleviation of drought and heat stress has focused on fungal endophytes. Hubbard et al. [63] showed that fungal endophytes improved wheat seed germination under heat-and drought-stress conditions. They also found that fungal endophytes were able to improve grain yield in heat-and drought-stress, as well as seed germination in second-generation seeds born from heat-stressed parents. Hamayun et al. [142] found that endophytic *Aspergillus japonicus* improved soybean and sunflower growth under heat-stressed conditions. Li et al. [143] and Zhang et al. [144] found that DSE improved host growth during drought stress through altered root development and phytohormone production, respectively. Ali et al. [145] inoculated fungal endophytes from delile (*Cullen plicata*) growing in hot desert soils onto heat-sensitive cucumbers, eliminating the adverse effects of heat stress. Similar drought stress amelioration effects in tomatoes were also achieved using endophytic *Neocamarosporium* spp. and *Periconia macrospinosa* from Hoz-e Soltan Salt Lake in Iran [146].

Research on the ability of desert plant endophytes to induce drought and heat resistance have focused on bacterial as well as fungal endophytes. Eke et al. [38] transferred endophytic bacteria from the cactus *Euphorbia trigonas* Mill to tomatoes, resulting in improved plantlet response to water stress. Zahra, Hamedi and Mahdigholi [147] inoculated sunflowers with *Streptomyces* spp. isolated from *Pteropyrum olivieri*, which increased seedling tolerance to drought stress.

Endophytes may be conferring heat and drought resistance in a variety of ways. One, the presence of microbial endophytes in the roots has been shown to alter root architecture, increasing lateral root development and the number of root hairs [12,42,148]. Changes in root architecture affect a plant’s ability to uptake water, which is especially important during periods of drought [149]. Second, the endophytes produce a variety of phytohormones, including indole-3-acetic acid (IAA), salicylic acid, ethylene, and gibberellic acid, which upregulate the plant’s abiotic stress resistance pathways [101,142,150,151]. Third, arbuscular mycorrhizae have been demonstrated to alleviate drought stress via the direct uptake and transfer of water via fungal hyphae [152,153], so it is quite likely that endophytic fungi are using a similar mechanism to alleviate drought stress in their hosts. There may also be additional ways in which endophytes confer resistance to drought and heat stress that have not yet been discovered.

There is a case to be made that crops can be planted in arid regions to naturally acquire drought-resistant microbes directly from the soil. Research by Marasco et al. [154] and Cherif et al. [38] has shown that plants cultivated in desert areas naturally acquire drought-resistant microbiomes. However, it is unknown if the microbiomes acquired by non-native plants are similar to those of native desert plants and whether there is a difference in the protections they offer to their hosts. In addition, it is important to keep in mind that attracting drought-resistant microbes may not be so easy. A study on the soil microbial community of semi-arid regions revealed that irrigation decreases the drought resistance of the soil microbiome [155], so croplands in arid and semi-arid areas may only harbor low amounts of drought-resistant microbes, or harbor drought-resistant microbes with diminished drought-stress-relieving properties. It may be more efficient to obtain drought-resistant microbes directly from wild plants growing in regions that are not irrigated in any way.

### 4.2. Salt Tolerance

Salt stress often accompanies drought stress, as the evaporation of water results in the concentration of salts in the soil. Most crop plants, including major crops such as wheat, corn, and rice, are glycophytes that cannot tolerate high levels of salt in the soil. Unlike animals, plants do not have Na^+^, K^+^-ATPases to pump excess Na+ out of their cells, and instead rely on a proton gradient to maintain proper levels of Na^+^ and K^+^ [156]. Excess Na^+^ in cells leads to many deleterious effects, such as metabolic dysregulation, nutrient uptake disruption, and loss of turgor pressure, which may then lead to plant death [157]. As such, high salt concentrations can be fatal to many plant species.

However, certain plants, such as those growing in mangroves and seashores, are halophytes that can tolerate high levels of salt in the soil. Their resistance to saline environments may be partially due to their endophytes-for instance, research by Rodriguez et al. [59] showed that fungal endophytes were required for salt tolerance in grasses native to saline coastal habitats. In addition, inoculating glycophytes with endophytes from halophytes has been shown to decrease the impacts of salt stress [158,159]. Most desert plants are categorized as halophytes, so their endophytes may also be able to confer resistance to high soil salinity.

Indeed, desert endophytes were able to improve host response to salt stress when inoculated into glycophytes. Trials on *Arabidopsis thaliana* showed that inoculation with *Bacillus* [44], *Enterobacter* [160], and *Athrobacter, Pantoea*, and *Microbacterium* [37] isolates from various desert plants showed improved resistance to salt stress and improved growth compared to non-inoculated controls. *Bacillus* spp. were shown to alleviate salt stress in tomatoes as well [161]. In addition, *Streptomyces* spp. isolated from *Pteropyrum olivieri* increased sunflower seedling tolerance to salt stress [147]. *Bacillus, Enterobacter, Pantoea, Microbacterium* and *Streptomyces* are all common bacterial endophytes of desert plants (Table 1), suggesting that more salt-resistance-conferring bacterial endophytes are yet to be discovered.

There is much less information available on the effects of desert endophytic fungi on host salt tolerance. Studies on barley showed that *Piriformospora indica*, an endophytic fungus isolated from the Thar Desert, was able to increase salt tolerance and growth in both salt-sensitive and salt-tolerant cultivars [137,162]. *Neocamarosporium* spp. and *Periconia macrospinosa* from the Hoz-e Soltan Salt Lake were shown to increase salt tolerance in cucumbers and tomatoes [146].

DSE have been shown to alleviate the symptoms of salt stress in glycophytes as well [163].

While there is some interest in using salt-resistance-conferring endophytes as growth promoting microbes, it is important to note that only some of these endophytes are able to promote host growth in normal conditions [146,147], while others are only able to promote host growth in saline environments [46,146,163]. It is unknown what causes the selectivity of these endophytes compared with other growth-promoting endophytes from desert plants, but it may be due to specialized pathways that only activate in high-saline environments.

The methods by which microbial endophytes influence salt tolerance is still not entirely known. Research performed by Eida et al. [37] suggest that bacterial endophytes may be increasing salt tolerance by altering the transcriptional regulation of ion transporters, thereby influencing the distribution of Na^+^ and K^+^ ions, while research by de Zélicourt et al. [160] suggests that production of a secondary metabolite is activating the ethylene salt response pathway. Other studies suggest that production of IAA [164,165,166] or ACC deaminase [167,168,169] are responsible for salt resistance instead. Since salt stress response is such a complex system, it is likely that all four mechanisms play some role in bacteria-mediated salt tolerance. Less is known about how fungal endophytes induce salt tolerance, but Baltruschat et al. [162] posits that it may be due to fungi-mediated increases in host antioxidant production. More research is needed to shed light on the mechanisms behind endophyte-mediated salt resistance in plants.

## 5. Effects on Biotic Stress Resistance

As the earth warms, pest and pathogen ranges and their lengths of activity are expected to expand [170,171,172]. This temporal and spatial expansion exposes more crops to novel pathogens and pests that they may not have resistance to, leading to dramatic yield losses. Currently, chemical pesticides and fungicides are the main solutions in combating pest and pathogen-induced yield losses, yet these are not sustainable long-term solutions due to off-target effects and the likelihood of agrochemical resistance development.

While deserts are generally known for their abiotic stressors, biotic stressors are still present. Pathogens such as Texas root rot (*Phymatotrichopsis omnivora*) and pests such as desert locusts exert selection pressure on native desert plants to acquire sources of resistance, such as endophytes, against their antagonists. Learning more about the endophytic microbiome of desert plants may allow researchers to find beneficial endophytes that can be used to adapt crops to biotic stressors unique to desert habitats, and perhaps to biotic stressors outside of deserts.

### 5.1. Pathogen Tolerance

The lack of water and nutrients in desert soils likely encourages symbiotic interactions between plants and microbes, particularly in the rhizosphere and root endophytic compartments. However, desert plants are still subject to pathogen attacks, even if such instances of such attacks may not be well documented in the wild. For instance, Texas root rot is a fungal pathogen that inhabits the alkaline desert soils of the southwestern United States and northern Mexico which can attack a variety of plants, mainly dicots [173]. Native desert dicots such as prickly pear cacti, desert willow, and palo verde are notably tolerant of the disease, perhaps partially due to the presence of their endophytes. Such disease pressure is likely present in other desert environments and researching the disease resistance capabilities of desert plants may produce novel solutions for growing non-native crops in desert environments that contain potent pathogens.

Several instances of desert endophytes conferring resistance to fungal pathogens have already been reported. Endophytic *P. indica* from the Thar Desert increased barley resistance to root pathogens [137], while *Bacillus* and *Enterobacter* from *Thymus vulgarius* in Egyptian deserts increased tomato resistance to *Fusarium oxysporum* [161].

Increased resistance to pathogen attack may simply be a byproduct of responses to abiotic stressors. Microbial endophytes produce many secondary metabolites such as IAA, ethylene, and giberillins that result in the upregulation of various plant pathways [101,174,175,176]. The upregulation of these plant pathways to combat abiotic stress may inadvertently affect pathways involved in plant defense. For instance, endophytes from the genus *Enterobacter* are known to produce a compound that is converted into ethylene, activating the ethylene-induced salt response pathway in plants [160]. Not only is ethylene involved in various aspects of plant growth and development, it is also involved in plant response to pathogen attack [177].

However, it is more likely that pathogen resistance comes from a combination of abiotic stress-related compounds and antimicrobial compounds. Endophytes are known to produce a variety of antimicrobial compounds, such as siderophores and lipopeptides [178,179,180,181]. In fact, certain desert endophytes have attracted attention for their antimicrobial properties [47,182,183]. There may be many more antimicrobial endophytes and compounds in desert plants that have not yet been discovered, as there are many instances of desert plants with medicinal properties [184,185] and research has shown that plants that produce more antimicrobial compounds have endophytes with more antimicrobial properties [186].

It may also be possible that endophytic bacteria in particular are colonizing the hyphae of pathogenic fungi, thus altering their pathogenicity. There have been reported instances of bacteria colonizing and parasitizing fungi [187,188,189]. In particular, bacteria from the family Proteobacteria, such as *Pseudomonas* and *Lysobacter*, have been shown to act as parasites of plant pathogenic fungi [190,191]. Based on the earlier meta-analysis, Proteobacteria make up the largest subset of endophytic bacteria in desert plants, so it may not be farfetched to assume that endophytic Proteobacteria also possess the ability to colonize and parasitize pathogenic fungi. More research would be needed to confirm or negate this hypothesis.

### 5.2. Pest Tolerance

According to the resource availability hypothesis (RAH), plants with low growth rates due to poor resource availability invest more into anti-herbivory defenses [192]. Deserts are some of the most resource-poor environments on Earth and contain some of the slowest growing plants on the planet, so desert plants should have many defenses against herbivores in accordance with the RAH.

One source of defense against herbivory comes from endophytes. There is evidence to suggest that plant resistance to insect damage is increased by the presence of both bacterial [193] and fungal [194,195] endophytes. Some studies on fungal desert endophytes demonstrate that they are able to increase host resistance and tolerance to herbivory: *P. indica* from the Thar Desert increases plant tolerance to root herbivory [196] and an *Epichloë* endophyte from a grass from the Sonoran Desert reduces seed harvesting by leaf cutter ants [197]. However, we are not aware of any published articles involving bacterial desert endophytes and their effect on herbivory. More research would be needed to find and document any bacterial endophytes that can reduce herbivory, particularly by insect pests.

Just as with endophyte-mediated pathogen resistance, endophyte-mediated pest resistance likely comes from a combination of secondary metabolite production and alteration of signaling pathways. In terms of secondary metabolite production, lipopeptides produced by bacteria can act as host defense inducers [198,199] or insecticidals [200], while the alkaloids produced by fungi are toxic to herbivores [194,201,202,203]. For instance, *Epichloë*, a known producer of alkaloids, is a fungal endophyte of grasses [202,203,204]. A study by Qin et al. [195] on *Achnatherum sibiricum*, a grass from the Mongolian steppe with inherently low alkaloid production, showed that infection with *Epichloë* endophytes reduced locust herbivory. Another study by Popay et al. [205] showed that *Epichloë* infection reduced scarab root herbivory of tall fescue. In terms of alteration of signaling pathways, endophytes are known to alter the production of various plant hormones in response to herbivory. Cosme et al. [196] exposed non-inoculated rice plants to weevils, which induced jasmonate signaling in the roots to suppress root growth. In plants inoculated with *P. indica,* jasmonate signaling was suppressed via gibberellic acid biosynthesis and root growth was recovered.

It is important to note that some fungal endophytes may rely on herbivores to infect other plants. Mechanical damage to leaves may facilitate the colonization of plant tissues, while spores and hyphae may be spread via gut excretions [65,66,67]; both of these methods of dispersal require herbivory, thus leading to endophyte-mediated decreases in host defenses. This encouragement of herbivory appears to be a characteristic of horizontally transmitted endophytes, while vertically transmitted endophytes are more likely to be toxic to herbivores [66]. More research is necessary to determine whether generalist endophytes from desert plants also follow this trend, or if they act more like host-specific endophytes due to the higher impact of herbivory on desert plants. It may be that endophytes of fast-growing but short-lived desert plants, such as *Alyssum alyssoides,* encourage herbivory, while the endophytes of slow-growing but long-lived desert plants, such as the *Cactaceae* and *Agavoideae*, discourage herbivory.

If it is found that desert endophytes, or at least a subset of them, act more like vertically transmitted endophytes while offering broad-host-spectrum protection against herbivorous pests, it may be worthwhile to introduce desert endophytes into crop plants to alleviate yield losses from insect damage.

## 6. Concluding Remarks

So far, the current published research around desert plant endophytes has revealed that they contribute to host fitness like endophytes found in other ecosystems. However, compared to endophytes from other regions, desert endophytes may be more relevant for agriculture now that climate change is creating extreme weather events that simulate desert-like conditions with increasing frequency. This combined with the population boom in arid regions has resulted in a need for plants that show increased resistance to abiotic stresses while still being able to produce high yields in order to match rising demands. Desert endophytes are known to increase host nitrogen and phosphorus acquisition, increase host tolerance to heat, water, and salt stress, as well as contribute to host biotic stress resistance. They tend to be generalists that can colonize and benefit a wide variety of hosts, which may be good when developing broad-range products for use in crops. However, this may come at the cost of reduced herbivory resistance, though there is no definitive evidence demonstrating if this principle applies to desert endophytes. Yet, the ability to provide all of the aforementioned benefits while being environmentally friendly makes desert endophytes stand out compared to traditional agrochemicals.

While some information has been elucidated about desert endophytes, our overall understanding of them is still poor. The mechanisms behind how they contribute to host survival is still relatively unknown, though some mechanisms can be extrapolated based on non-desert endophyte interactions with their hosts. Overall, there are still questions that have yet to be answered regarding desert endophytes and endophytes in general.

For one, interactions between endophytes, particularly how fungal and bacterial endophytes interact with one another, is poorly understood. There is evidence to suggest that bacterial and fungal endophytes may work together synergistically to improve plant growth–studies by Khan et al. [206] and Yousefi et al. [207] showed that phosphate solubilizing bacteria had greater solubilization efficiency when combined with certain other endophytes and mycorrhizae. A study by Bandara, Seneviratne & Kulasooriya [208] revealed that biofilms formed by endophytic bacteria and fungi secreted higher levels of IAA-like substances compared to either endophytic bacteria or endophytic fungi alone. However, biofilms may not necessarily form in planta, as the physical architecture of the host tissues can act as physical barriers to prevent the interaction of endophytic microbes. Conversely, there is also evidence to suggest that bacterial and fungal endophytes may work antagonistically to suppress one another. Araújo et al. [209] showed that *Guignardia citricarpa*, a fungal endophyte, suppresses *Bacillus* endophytes while stimulating *Pantoea agglomerans* endophytes. It is likely that there are specific combinations of endophytes that can interact synergistically to create maximum gains in host nutrient acquisition or stress resistance, but such compatibility studies have not been performed on a wide scale. Desert endophytes in particular may have special interactions with one another due to high degrees of colonization and high host generalism.

Another topic that should be explored further is the difference in nutrient acquisition and stress resistance between desert and non-desert endophytes. Are there notable differences between the two groups, and in which conditions? It would be expected that desert endophyte-inoculated plants would fare better in desert-like environments, while non-desert endophyte-inoculated plants would fare better in their native environments; however, any deviations from the expected results would teach us more about how microbiome composition affects plant responses to different environmental conditions, especially if it turns out that both groups perform similarly.

As the agricultural industry is highly influenced by consumer spending habits, public acceptance is important to the implementation of any new technologies. Fortunately, biocontrol agents and biofertilizers fall in line with current consumer trends, which show a shift away from foods produced using agrochemicals and towards organic foods produced without using agrochemicals [25,26,27]. Not only that, public perception of agrochemicals, particularly of pesticides and herbicides, is becoming increasingly negative [28,29,30], an issue which is compounded by various lawsuits that have been filed against agricultural companies in regard to the health risks associated with pesticide and herbicide applications [210,211,212]. Companies that are developing microbe-based alternatives to traditional agrochemical solutions may begin to see more support from the public, as well as from farmers who are appealing to the organic market.

In all, desert endophytes show promise as biofertilizers, biocontrol agents, and abiotic stress relievers of crops, particularly ones grown in arid regions. This is especially important as crops face increasing stressors from climate change and the agricultural industry faces mounting pressure to break away from agrochemicals towards more environmentally friendly alternatives. There is still much to be discovered about desert endophytes, which may be uncovered with further research.

## Figures and Tables

**Table 1:** Breakdown of culturable bacterial endophyte isolates by genus. References: [36,37,38,39,40,41,42,43,44,45,46,47].

Phylum	Genus	# of Isolates	% of Phylum	% of All Bacterial Endophytes
Actinobacteria	*Microbacterium*	91	51.70%	12.69%
	*Micrococcus*	6	3.41%	0.84%
	*Arthrobacter*	6	3.41%	0.84%
	*Streptomyces*	20	11.36%	2.79%
	*Cellulomonas*	31	17.61%	4.32%
	*Citrococcus*	1	0.57%	0.14%
	*Curtobacterium*	3	1.70%	0.42%
	*Kocuria*	1	0.57%	0.14%
	*Promicromonospora*	1	0.57%	0.14%
	*Aeromicrobium*	2	1.14%	0.28%
	*Amycolatopsis*	3	1.70%	0.42%
	*Cellulosimicrobium*	1	0.57%	0.14%
	*Corynebacterium*	1	0.57%	0.14%
	*Gordonia*	3	1.70%	0.42%
	*Herbiconiux*	1	0.57%	0.14%
	*Leucobacter*	1	0.57%	0.14%
	*Kineococcus*	1	0.57%	0.14%
	*Mycobacterium*	1	0.57%	0.14%
	*Nonomuraea*	1	0.57%	0.14%
	*Spirillospora*	1	0.57%	0.14%
α-Proteobacteria	*Gluconobacter*	33	27.97%	4.60%
	*Agrobacterium*	14	11.86%	1.95%
	*Rhizobium*	13	11.02%	1.81%
	*Mesorhizobium*	11	9.32%	1.53%
	*Inquilinus*	8	6.78%	1.12%
	*Sinorhizobium/Ensifer*	8	6.78%	1.12%
	*Azospirillum*	7	5.93%	0.98%
	*Paracoccus*	4	3.39%	0.56%
	*Sphingomonas*	4	3.39%	0.56%
	*Unknown genus*	4	3.39%	0.56%
	*Devosia*	3	2.54%	0.42%
	*Brevundimonas*	2	1.69%	0.28%
	*Methylobacterium*	2	1.69%	0.28%
	*Altererythrobacter*	1	0.85%	0.14%
	*Miroviga*	1	0.85%	0.14%
	*Ochrobactrum*	1	0.85%	0.14%
	*Rhodobacter*	1	0.85%	0.14%
	*Roseomonas*	1	0.85%	0.14%
β-Proteobacteria	*Cupriavidus*	20	54.05%	2.79%
	*Variovorax*	7	18.92%	0.98%
	*Ralstonia*	3	8.11%	0.42%
	*Massilia*	2	5.41%	0.28%
	*Achromobacter*	1	2.70%	0.14%
	*Bordetella*	1	2.70%	0.14%
	*Burkholderia*	1	2.70%	0.14%
	*Thibacillus*	1	2.70%	0.14%
	*Tetrathiobacter*	1	2.70%	0.14%
γ-Proteobacteria	*Pseudomonas*	53	28.96%	7.39%
	*Acinetobacter*	37	20.22%	5.16%
	*Enterobacter*	24	13.11%	3.35%
	*Pantoea*	14	7.65%	1.95%
	*Stenotrophomonas*	11	6.01%	1.53%
	*Cronobacter*	11	6.01%	1.53%
	*Erwinia*	8	4.37%	1.12%
	*Klebsiella*	7	3.83%	0.98%
	*Serratia*	6	3.28%	0.84%
	*Buttiauxella*	2	1.09%	0.28%
	*Citrobacter*	2	1.09%	0.28%
	*Halomonas*	2	1.09%	0.28%
	*Rahnella*	2	1.09%	0.28%
	*Aeromonas*	1	0.55%	0.14%
	*Azotobacter*	1	0.55%	0.14%
	*Lelliottia*	1	0.55%	0.14%
	*Proteus*	1	0.55%	0.14%
Firmicutes	*Bacillus*	148	78.72%	20.64%
	*Paenibacillus*	17	9.04%	2.37%
	*Brevibacillus*	5	2.66%	0.70%
	*Staphylococcus*	5	2.66%	0.70%
	*Rumeliibacillus*	3	1.60%	0.42%
	*Planomicrobium*	2	1.06%	0.28%
	*Enterococcus*	1	0.53%	0.14%
	*Exiguobacterium*	1	0.53%	0.14%
	*Leuconostoc*	1	0.53%	0.14%
	*Lysinibacillus*	1	0.53%	0.14%
	*Planococcus*	2	1.06%	0.28%
	*Saccharibacillus*	1	0.53%	0.14%
	*Streptococcus*	1	0.53%	0.14%
Bacteroidetes	*Sphingobacterium*	7	46.67%	0.98%
	*Chryseobacterium*	4	26.67%	0.56%
	*Olivibacter*	3	20.00%	0.42%
	*Algoriphagus*	1	6.67%	0.14%
Totals		717		100%

**Table 2:** Breakdown of culturable fungal endophyte isolates by phyla, class, and subclass. References: [51,52,53,54,55,56,57].

Phylum	Subphylum	Class	Genus	# of Isolates	% of Class	% of Phylum	% of Total
Ascomycota	Pezizomycotina	Dothideomycetes	*Alternaria*	476	30.75%	21.29%	18.89%
			*Phoma*	444	28.68%	19.86%	17.62%
			*Aureobasidium*	242	15.63%	10.82%	9.60%
			*Cladosporium*	150	9.69%	6.71%	5.95%
			*Ascochyta*	95	6.14%	4.25%	3.77%
			*Coniothyrium*	37	2.39%	1.66%	1.47%
			*Epicoccum*	36	2.33%	1.61%	1.43%
			*Leptosphaeria*	16	1.03%	0.72%	0.63%
			*Boeremia*	1	0.07%	0.05%	0.04%
			*Cochliobolus*	7	0.45%	0.31%	0.28%
			*Stemphylium*	7	0.45%	0.31%	0.28%
			*Curvularia*	6	0.39%	0.26%	0.24%
			*Preussia*	6	0.39%	0.266%	0.24%
			*Drechslera*	5	0.32%	0.22%	0.20%
			*Embellisia*	4	0.26%	0.18%	0.16%
			*Macrophomina*	3	0.19%	0.13%	0.12%
			*Guignardia*	2	0.13%	0.09%	0.08%
			*Aerobasidium*	1	0.07%	0.05%	0.04%
			*Paraconiothyrium*	2	0.13%	0.09%	0.08%
			*Paraphoma*	2	0.13%	0.09%	0.08%
			*Torula*	2	0.13%	0.09%	0.08%
			*Unocladium*	2	0.13%	0.09%	0.08%
			*Pseudocochliobolus*	1	0.07%	0.09%	0.04%
			Unknown Pleosporales	1	0.07%	0.09%	0.04%
		Sordariomycetes	*Fusarium*	131	27.01%	5.86%	5.20%
			*Nigrospora*	57	11.75%	2.55%	2.26%
			*Acremonium*	54	11.13%	2.42%	2.14%
			*Chaetomium*	41	8.45%	1.83%	1.63%
			*Coniella*	40	8.25%	1.79%	1.59%
			*Trichoderma*	27	5.57%	1.21%	1.07%
			*Monosporascus*	23	4.74%	1.03%	0.91%
			*Sordaria*	17	3.51%	0.76%	0.67%
			*Chrysonilia*	15	3.09%	0.67%	0.60%
			*Diaporthe*	15	3.09%	0.67%	0.60%
			*Cytospora*	14	2.89%	0.63%	0.56%
			*Pestalotiopsis*	9	1.86%	0.40%	0.36%
			*Geniculosporium*	8	1.65%	0.36%	0.32%
			*Nodulisporium*	8	1.65%	0.36%	0.32%
			*Gibberella*	7	1.44%	0.31%	0.28%
			*Phomopsis*	7	1.44%	0.31%	0.28%
			*Sarocladium*	3	0.62%	0.13%	0.12%
			*Bartalinia*	1	0.21%	0.05%	0.04%
			*Biscogniauxia*	1	0.21%	0.05%	0.04%
			*Coniochaeta*	1	0.21%	0.05%	0.04%
			*Myrothecium*	1	0.21%	0.05%	0.04%
			*Nectria*	1	0.21%	0.05%	0.04%
			*Neonectria*	1	0.21%	0.05%	0.04%
			*Plectosphaerella*	1	0.21%	0.05%	0.04%
			*Purpureocillium*	1	0.21%	0.05%	0.04%
			Unk. Coniochaetales	1	0.21%	0.05%	0.04%
		Eurotiomycetes	*Penicillium*	134	71.28%	5.99%	5.32%
			*Aspergillus*	51	27.13%	2.28%	2.02%
			*Phinocladiella*	3	1.60%	0.13%	0.12%
		Leotiomycetes	*Cadophora*	1	50.00%	0.05%	0.04%
			*Phialocephala*	1	50.00%	0.05%	0.04%
	Saccharomycotina	Saccharomycetes	*Debaryomyces*	6	75.00%	0.27%	0.24%
			*Candida*	2	25.00%	0.09%	0.08%
	Incertae sedis	Incertae sedis	*Rhizopycnis*	4	80.00%	0.18%	0.16%
			*Aporospora*	1	20.00%	0.05%	0.04%
Zygomycota	n/a	Zygomycetes	*Mucor*	9	42.86%	42.86%	0.36%
			*Rhizopus*	8	38.10%	38.10%	0.32%
			*Cunninghamella*	3	14.29%	14.29%	0.12%
			*Syncephalastrum*	1	4.76%	4.76%	0.04%
Basidiomycota	Agaricomycotina	Agaricomycetes	*Rhizoctonia*	1	100.00%	5.26%	0.04%
		Tremeliomycetes	*Cryptococcus*	1	100.00%	5.26%	0.04%
	Pucciniomycotina	Agaricostilbomycetes	*Sterigmatomyces*	5	100.00%	26.32%	0.20%
		Microbotryomycetes	*Rhodotorula*	6	75.00%	31.58%	0.24%
			*Sporobolomyces*	2	25.00%	10.53%	0.08%
		Tritirachiomycetes	*Tritirachium*	4	100.00%	21.05%	0.16%
Sterile	Unk.	Unknown	Unknown	244	100.00%	100.00%	9.68%
		Total		2520			100%

## Data Availability

Not applicable.
